# Uncertainty analysis of physical-based carbon accounting in cotton T-shirt manufacturing

**DOI:** 10.1038/s41598-026-38773-4

**Published:** 2026-02-06

**Authors:** Emmanuel Olugbemi, Natanael Favero Bolson

**Affiliations:** School of Engineering, University of Birmingham, Birmingham, B15 2TT UK

**Keywords:** Carbon accounting, Life cycle assessment, Uncertainty analysis, Pedigree matrix, Cotton T-shirt manufacturing, Climate sciences, Energy science and technology, Engineering, Environmental sciences, Environmental social sciences, Mathematics and computing

## Abstract

The textile sector contributes significantly to global greenhouse gas emissions, yet product-level carbon accounting in this industry remains constrained by data gaps and methodological uncertainty. This study quantifies the uncertainty embedded in process-based carbon accounting for the cradle-to-gate production of a cotton T-shirt. We evaluated secondary activity data and emission factors using a pedigree matrix across five data quality indicators: precision, completeness, and temporal, geographical, and technological representativeness. The total cradle-to-gate carbon footprint was ${1.37}\,\hbox {kg}\,\hbox {CO}_{2}\hbox {e}$ per T-shirt, with an overall uncertainty of ±13.81 %. Fabric production was the largest source of emissions and uncertainty, contributing over 60 % of total emissions and nearly 70 % of total uncertainty, driven by energy-intensive processes such as weaving, dyeing, and sanforising. Yarn spinning was a secondary hotspot, while the sewing stage had comparatively minor impacts. The results show significant variation in data reliability across production stages and highlight the need for improved primary data, updated emission factors, and harmonised databases to strengthen the robustness and comparability of process-based carbon accounting in the textile sector.

## Introduction

The textile industry is a significant contributor to global greenhouse gas (GHG) emissions, accounting for approximately 10 % of worldwide emissions^1^. Within this sector, cotton production is among the most resource- and energy-intensive activities. Cotton cultivation alone is estimated to contribute around 60 % of the total carbon footprint of a T-shirt, primarily due to fertiliser use, irrigation practices, and their associated emissions^2,3^. Subsequent stages such as spinning, weaving, dyeing, and finishing also demand substantial electricity and material inputs, making their emissions highly sensitive to regional energy mixes and technological configurations^4^. The rise of fast fashion further aggravates these impacts by accelerating production cycles and increasing overall resource consumption^5^.

Carbon accounting provides a systematic framework to quantify, report, and manage emissions across the life cycle of products. Two main approaches dominate: the spend-based approach, which applies environmental input–output models to monetary data, and the physical-based approach, which uses process-specific activity data such as kilowatt-hours of electricity^6^. Spend-based methods are cost-effective and useful for broad estimates, but they lack precision because they rely on aggregated sector averages and are sensitive to inflation and misclassification errors^7,8^. In contrast, physical-based accounting aligns with life cycle assessment (LCA) methodologies and provides greater traceability across production stages, making it more suitable for detailed product-level analysis^2,9^. Comparative studies have shown that physical-based methods offer greater traceability and sensitivity to technological and energy-system differences, particularly when assessing upstream emissions at the process level^10^.

In cotton T-shirt manufacturing, uncertainty arises from variability in data quality, model assumptions, and parameter ranges^11^. Additional sources of uncertainty stem from differences in agricultural practices, regional electricity mixes, and technological variation across factories, all of which affect the accuracy of carbon footprint estimates^12^. The contribution of individual parameters to total uncertainty depends on their uncertainty range and model sensitivity, such that highly sensitive parameters can dominate overall uncertainty even when their nominal uncertainty is small^13^. To systematically assess these uncertainties, the pedigree matrix has become a widely used framework for evaluating data quality across five dimensions: precision, completeness, temporal representativeness, geographical representativeness, and technological representativeness^14,15^. Qualitative ratings for each criterion are translated into quantitative uncertainty factors, which are then aggregated using statistical methods to generate confidence intervals instead of single-point estimates^16^.

This study focuses exclusively on the physical-based approach to carbon accounting for cotton T-shirt manufacturing. It aims to quantify the uncertainty associated with cradle-to-gate emission estimates by applying the pedigree matrix across five data quality indicators. In doing so, the study examines how modelled uncertainty varies across precision, completeness, temporal representativeness, geographical representativeness, and technological representativeness when using physical-based methods. By addressing these aspects, the study advances methodological transparency in product carbon accounting and offers practical guidance to improve the credibility of emission estimates in the textile sector.

We apply a process-based approach to cotton T-shirt manufacturing and: (i) quantify cradle-to-gate emissions, (ii) evaluate how uncertainty is distributed across production stages and processes using the pedigree matrix, and (iii) assess the influence of technological variants. The findings provide targeted recommendations to enhance data quality and improve comparability in textile carbon accounting.

## Methodology

**Fig. 1:**
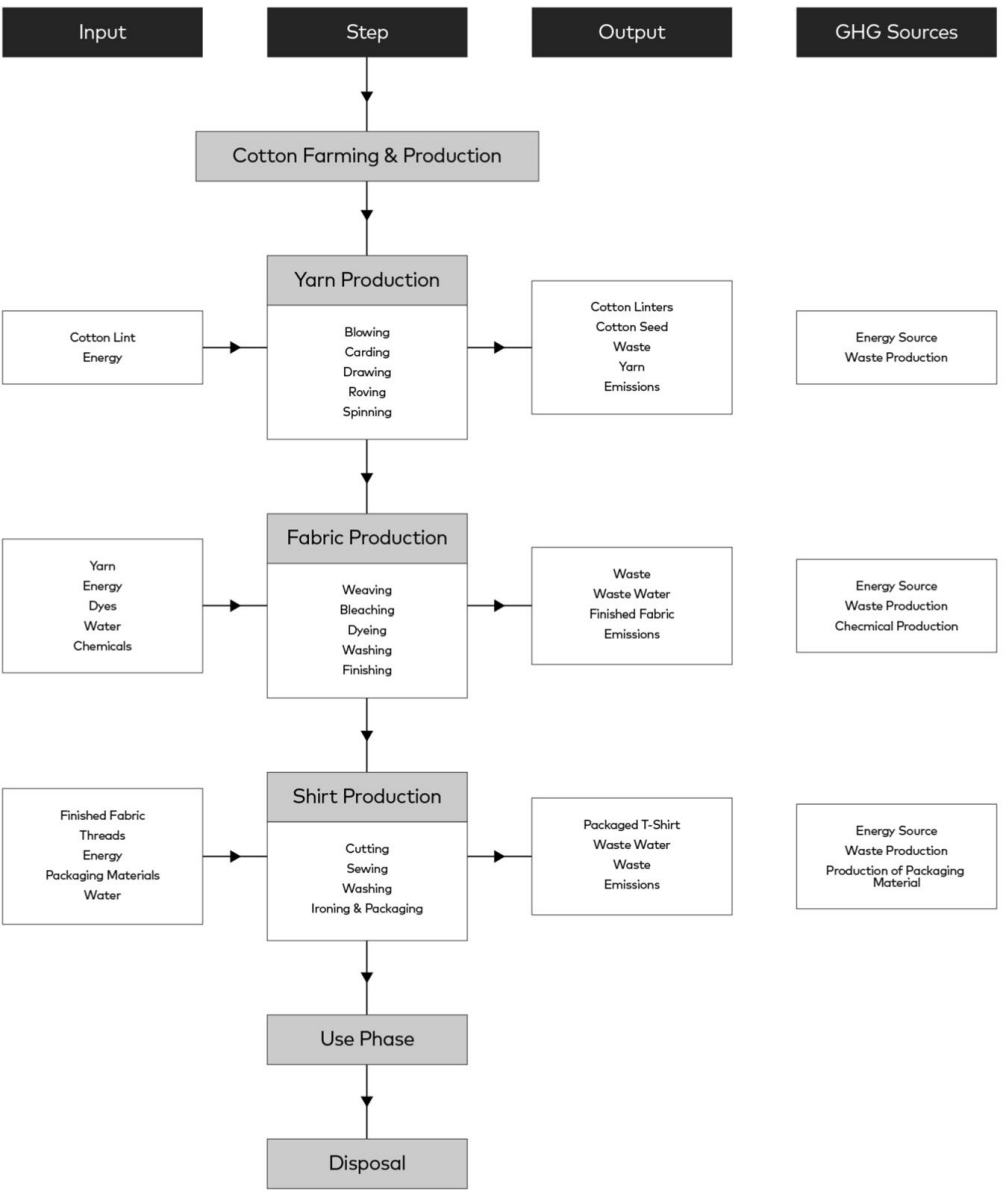
Process flow and system boundary for the cradle-to-gate assessment of a 150 g cotton T-shirt. Included processes are yarn production (blowing, carding, drawing, roving, and spinning), fabric production (weaving, bleaching, dyeing, and finishing), and T-shirt production (cutting, sewing, washing, ironing and packaging). Excluded processes are cotton cultivation, transportation between stages, equipment, maintenance, and capital goods.

This study employs a quantitative life cycle assessment (LCA) framework with a focus on uncertainty analysis. A physical-based carbon accounting approach is adopted, relying on process-specific activity data and corresponding emission factors. The functional unit is a 100 % cotton T-shirt with an average mass of 150 g^17^. The system boundary is defined as cradle-to-gate and includes yarn production (blowing, carding, drawing, roving, and spinning), fabric production (weaving, bleaching, dyeing, and finishing), and T-shirt production (cutting, sewing, washing, ironing and packaging). Cotton cultivation, transportation between production stages, capital goods, and the construction and maintenance of facilities and equipment are excluded. Figure 1 illustrates the process flow and system boundary considered in the analysis, highlighting the inputs, three main production stages, associated sub-processes, outputs, and GHG sources.

Data for this study were sourced exclusively from secondary activity data and emission intensity (emission factor) datasets available in free LCA databases^18^, peer-reviewed literature^19–22^, and official reports^23–28^. Activity data and emission factors were compiled to develop the inventory, and datasets were selected, where possible, to reflect cotton-based textile production in comparable geographical and technological contexts. All activity data originally provided in MJ/kg or MJ/t were converted to kWh/kg.

Material yield was incorporated into the assessment of T-shirt manufacturing. T-shirt production was assumed to have a material efficiency of 90 %^19^, while fabric manufacturing efficiencies reported in the literature range from 96 % to 99 %^23–29^. For this study, a midpoint value of 97.5 % was adopted.

The pedigree matrix scoring method was applied to the collected data to evaluate its quality across five indicators: precision (reliability of measured or estimated values), completeness (coverage of relevant flows), temporal representativeness (age of the data), geographical representativeness (regional relevance), and technological representativeness (appropriateness of the applied technology). Each indicator was scored on a defined scale and then converted into percentage uncertainty ranges using established pedigree conversion formulas. The scoring approach follows the guidance of the GHG Protocol guidance for uncertainty^15^. Table 1 presents the scoring framework and criteria used.

**Table 1:** Pedigree matrix indicators and qualitative anchors. Top rows: uncertainty factors used to convert qualitative scores to multiplicative spreads. Bottom rows: qualitative anchors for scoring.

Indicator	Very good	Good	Fair	Poor
**Uncertainty factor**				
Precision	1.00	1.10	1.20	1.50
Completeness	1.00	1.05	1.10	1.20
Temporal representativeness	1.00	1.10	1.20	1.50
Geographical representativeness	1.00	1.02	1.05	1.10
Technological representativeness	1.00	1.20	1.50	2.00
**Qualitative anchors**				
Precision	Measured, high confidence	Literature estimate, clear basis	Expert judgement	Assumed/educated guess
Completeness	>90 % flows	75 % to 90 %	55 % to 75 %	<55 %, key flows missing
Temporal rep.	<3 years old	3–6 years	6–10 years	>10 years
Geographical rep.	Exact site/region	Country	Continent	Global average
Technological rep.	Major technology	Similar	Older/uncertain	Different/unknown

Uncertainty propagation was performed using an analytical, pedigree-matrix–based approach in accordance with GHG Protocol guidance, rather than Monte Carlo simulation.

Uncertainty calculations followed the GHG Protocol guidance on uncertainty assessment in greenhouse gas inventories and statistical parameter uncertainty^30^, as well as the Greenhouse Gas Protocol’s Quantitative Inventory Uncertainty document^15^. Using the pedigree matrix scores for each data point (both activity data and emission factors), total uncertainty was quantified as the square of the geometric standard deviation, calculated using the formula below:

1 $$\begin{aligned} \textrm{SD} = \exp \!\left( \sqrt{\sum _{k=1}^{5}[\ln (U_k)]^2}\right) \end{aligned}$$

$$\begin{aligned} k \in \{\text {precision, completeness, temporal, geographical, technological}\}. \end{aligned}$$

Once the total uncertainty values were derived for activity data and emission factors, step uncertainty was calculated using two different approaches, depending on the percentage uncertainty of the data, in accordance with GHG Protocol guidance^30^. Process-level uncertainty is then:

2 $$\begin{aligned} U_{\text {step}} = {\left\{ \begin{array}{ll} \sqrt{U_{\text {AD}}^{\,2}+U_{\text {EF}}^{\,2}}, & \text {if } U<{60}\,{\%},\\ U_{\text {AD}} + U_{\text {EF}}, & \text {otherwise.} \end{array}\right. } \end{aligned}$$

Where $U_{\text {AD}}$ is the uncertainty of the activity data, and $U_{\text {EF}}$ is the uncertainty of the emission factor.

With the step uncertainty obtained, the stage uncertainty is determined using the formula below:

3 $$\begin{aligned} U_{\text {stage}} = \frac{\sqrt{\sum (U_i \cdot E_i)^2}}{\sum E_i} \end{aligned}$$

Where $U_i$ is the uncertainty of each step, and $E_i$ is the calculated emission of that step.

To calculate the overall uncertainty for the T-shirt, a case study was constructed using emission and uncertainty values from India (2015) for yarn and fabric production^23,27^ and from China (2015) for T-shirt manufacturing^20,22^. The combined uncertainty was then calculated using the following formula:

4 $$\begin{aligned} U_{\text {T-shirt}} = \frac{\sqrt{\sum (U_{si} \cdot E_{si})^2}}{\sum E_{si}} \end{aligned}$$

For analysis, further calculations were done to determine the percentage uncertainty and emission contribution of each step using the formulas below:

5 $$\begin{aligned} & U{_\text {Contribution}}~(\%) = \frac{(U_i \cdot E_i)^2}{\sum (U_i \cdot E_i)^2}\times 100 \end{aligned}$$

6 $$\begin{aligned} & E{_\text {Contribution}}~(\%) = \frac{E_i}{\sum E_i}\times 100 \end{aligned}$$

## Results and discussions

### Emissions

**Fig. 2:**
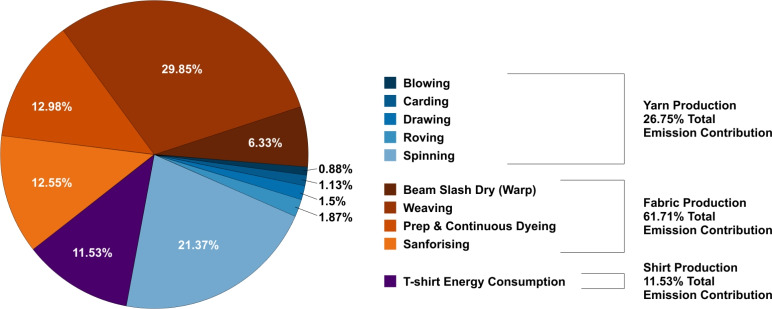
Percentage contributions to the total cradle-to-gate footprint by stage and by process. The functional unit is a cotton T-shirt. Reference year 2015.

Figure 2 presents the percentage contributions of each production stage and sub-process to the total cradle-to-gate carbon footprint of a 150 g cotton T-shirt. The overall footprint was estimated at ${1.3706}\,\hbox {kg}\,\hbox {CO}_{2}\hbox {e}$. Fabric production was the largest contributor, responsible for ${0.8459}\,\hbox {kg}\,\hbox {CO}_{2}\hbox {e}$, or 61.71 % of the total. Within this stage, weaving accounted for 29.85 % of total emissions, followed by dyeing (12.98 %) and sanforising (12.55 %). Yarn production was the second-largest contributor: spinning alone made up 21.37 % of total emissions, while blowing, carding, drawing, and roving collectively contributed 5.38 %. These process-level emission profiles also align with the distribution of uncertainty, as the most energy-intensive stages, particularly weaving and spinning, exert the strongest influence on the overall uncertainty of the footprint.

**Fig. 3:**
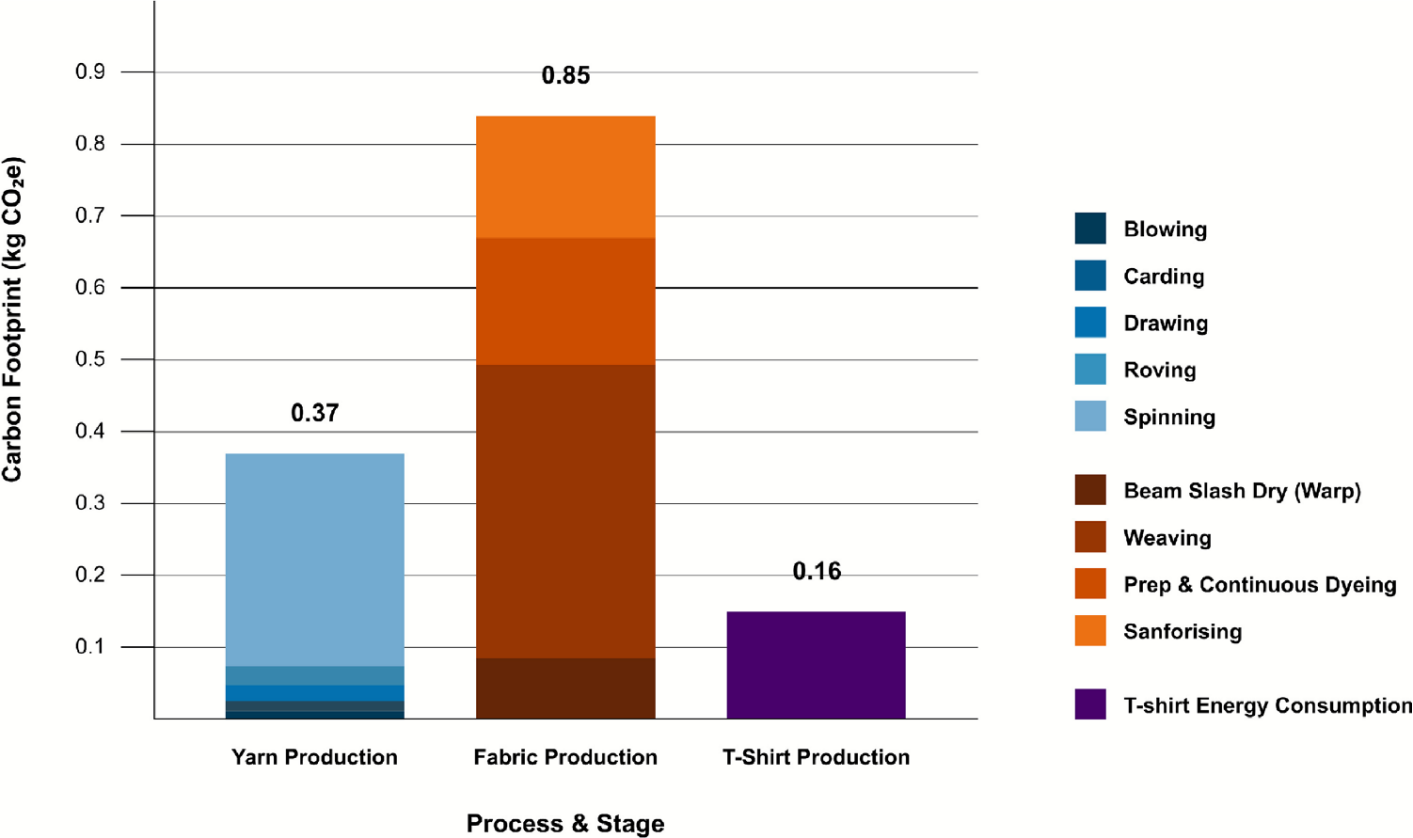
Absolute emissions by stage with breakdown by process. Bars show mean values. Fabric production exceeds the combined emissions of yarn and sewing.

These results reinforce fabric production as the principal source of emissions and align with previous LCA studies that identify wet processes such as dyeing, bleaching, and finishing as particularly carbon-intensive due to their high electricity and chemical requirements. Jewell et al. reported textile manufacturing to account for approximately 54 % of Global Warming Potential (GWP) and 58 % of Primary Energy Demand (PED)^23^. Similarly, Barnes et al. found textile manufacturing to be the second most impactful stage (after the use phase) across multiple categories, including GWP, Acidification Potential (AP), Eutrophication Potential (EP), Ozone Depletion Potential (ODP), and Photochemical Ozone Creation Potential (POCP)^31^. Figure 3 presents the total emissions by process and stage, showing fabric production at ${0.85}\,\hbox {kg}\,\hbox {CO}_{2}\hbox {e}$, exceeding the combined emissions of yarn production (${0.37}\,\hbox {kg}\,\hbox {CO}_{2}\hbox {e}$) and T-shirt production (${0.16}\,\hbox {kg}\,\hbox {CO}_{2}\hbox {e}$), which together amount to ${0.53}\,\hbox {kg}\,\hbox {CO}_{2}\hbox {e}$.

The full calculation table is provided in Table S1 of the Supplementary Information.

## Uncertainty

To assess data quality, the pedigree scoring method was applied to the activity data and emission factors for each process, following the framework presented in Table 1. The pedigree scores for all stages and processes of the T-shirt are provided in Table S2 of the Supplementary Information.

These scores were translated into percentage uncertainty values and then aggregated to derive process-level uncertainties. As shown in Table 2, uncertainty is not evenly distributed across processes or stages. In both the yarn and fabric production stages, activity data dominate the uncertainty, with activity data uncertainty values of 32.30 % and emission factor uncertainty of only 2 %, resulting in a consistent step uncertainty of 32.36 % across individual processes. However, their relative contributions vary substantially. In yarn production, spinning alone accounts for over 98 % of the stage-level uncertainty, while all other processes in that stage (blowing, carding, drawing, and roving) together contribute less than 2 %. A similar pattern is observed in fabric production, where weaving contributes nearly 71 % of the stage-level uncertainty, followed by prep and continuous dyeing (13.40 %) and sanforising (12.53 %), with beam slash dry having a minor influence. In contrast, T-shirt production is dominated entirely by sewing, which shows a lower activity data uncertainty of 23.66 % but a comparatively higher emission factor uncertainty of 14.60 %, resulting in a step uncertainty of 27.80 % and accounting for 100 % of the uncertainty in that stage.

**Table 2:** Process-level percentage uncertainties and contribution to stage-level uncertainty.

Process (step)	U$_{\text {AD}}$(%)	U$_{\text {EF}}$(%)	U$_{\text {step}}$(%)	Contribution (%)
**Yarn Production**				
Blowing	32.30	2.00	32.36	0.17
Carding	32.30	2.00	32.36	0.28
Drawing	32.30	2.00	32.36	0.48
Roving	32.30	2.00	32.36	0.75
Spinning	32.30	2.00	32.36	98.32
**Fabric Production**				
Beam slash dry (warp)	32.30	2.00	32.36	3.19
Weaving	32.30	2.00	32.36	70.88
Prep & continuous dyeing	32.30	2.00	32.36	13.40
Sanforising	32.30	2.00	32.36	12.53
**T-shirt Production**				
Sewing energy	23.66	14.60	27.80	100.00

**Fig. 4:**
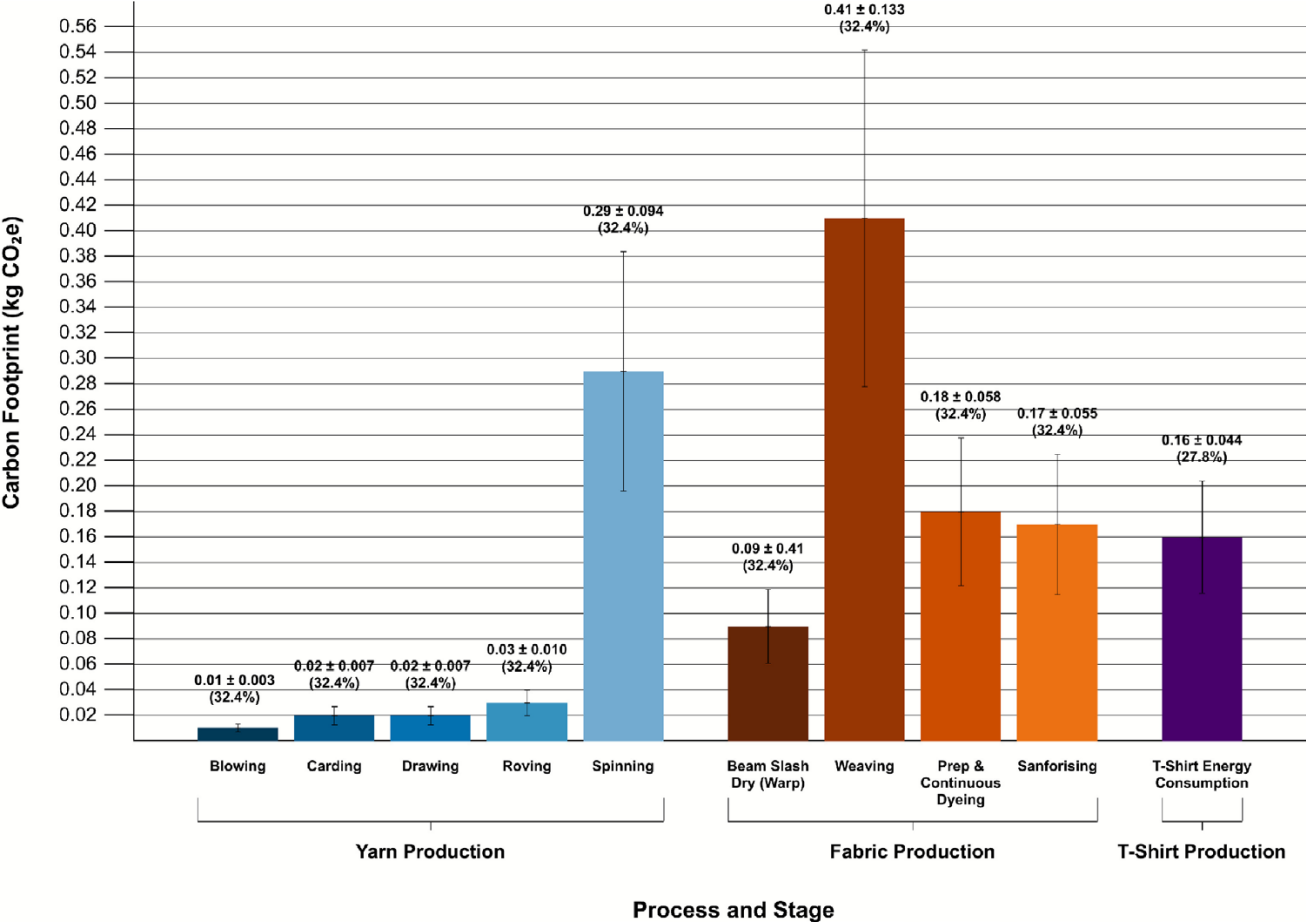
Process-level emissions with propagated uncertainty. Bars show mean emissions per process, and error-bars show the standard deviation.

Although the yarn and fabric production stages share the same data sources and therefore exhibit identical process-level percentage uncertainties, these values do not translate proportionally into their contributions to stage- or product-level uncertainty. This is because the magnitude of emissions in each process influences its overall impact. Figure 4 presents the emissions alongside their associated uncertainty ranges for each process. The weaving process has the highest absolute uncertainty, at $\pm 0.1333\,\hbox {kg}\hbox {CO}_{2}\hbox {e}$, reflecting both its high emissions and its contribution to stage-level uncertainty. By contrast, the blowing process has the lowest absolute uncertainty, at $\pm 0.003\,\hbox {kg}\hbox {CO}_{2}\hbox {e}$, due to its comparatively minor emissions.

Stage-level uncertainties were then derived by aggregating the process-level results. The yarn production stage showed an uncertainty of 26.08 %, followed by fabric production at 18.59 %, and T-shirt production at 27.80 %. Although fabric production is the largest contributor to total emissions, its relative uncertainty is lower because the underlying activity data and emission factors are more consistently reported across sources. These values are summarised in Table 3, which also reports the total emissions and squared weighted uncertainty terms for each stage.

**Table 3:** Stage-level uncertainty summary.

Stage	Process emissions ($\hbox {kg}\hbox {CO}_{2}\hbox {e}$)	$\mathbf {\sum (U\,\times \,\textrm{GHG})^2}$	Stage uncertainty (%)
Yarn production	0.3667	0.0091	26.08
Fabric production	0.8459	0.0247	18.59
T-shirt production	0.1581	0.0019	27.80

**Fig. 5:**
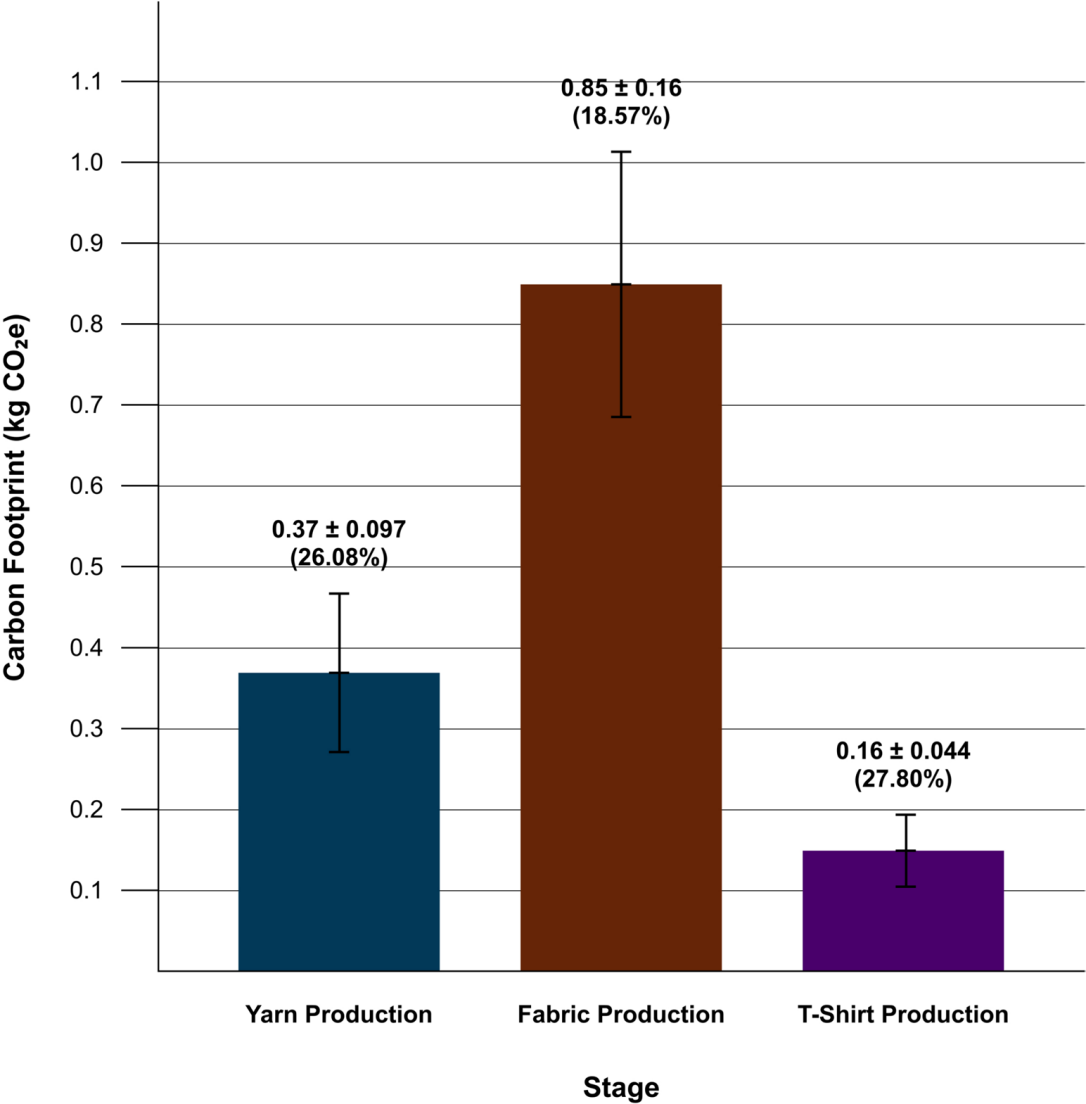
Stage emissions with percentage uncertainty bands (shaded). Although fabric has the lowest percentage uncertainty, it dominates absolute uncertainty due to its emission magnitude.

Figure 5 presents the stage-level emissions alongside their corresponding uncertainty ranges. The fabric production stage has the lowest percentage uncertainty at 18.57 %, but due to its emission magnitude, it still exhibits the highest absolute emission uncertainty of $\pm 0.016\,\hbox {kg}\hbox {CO}_{2}\hbox {e}$. By contrast, T-shirt production has the highest percentage uncertainty at 27.80 %, yet the lowest absolute emission uncertainty of $\pm 0.044\,\hbox {kg}\hbox {CO}_{2}\hbox {e}$.

**Fig. 6:**
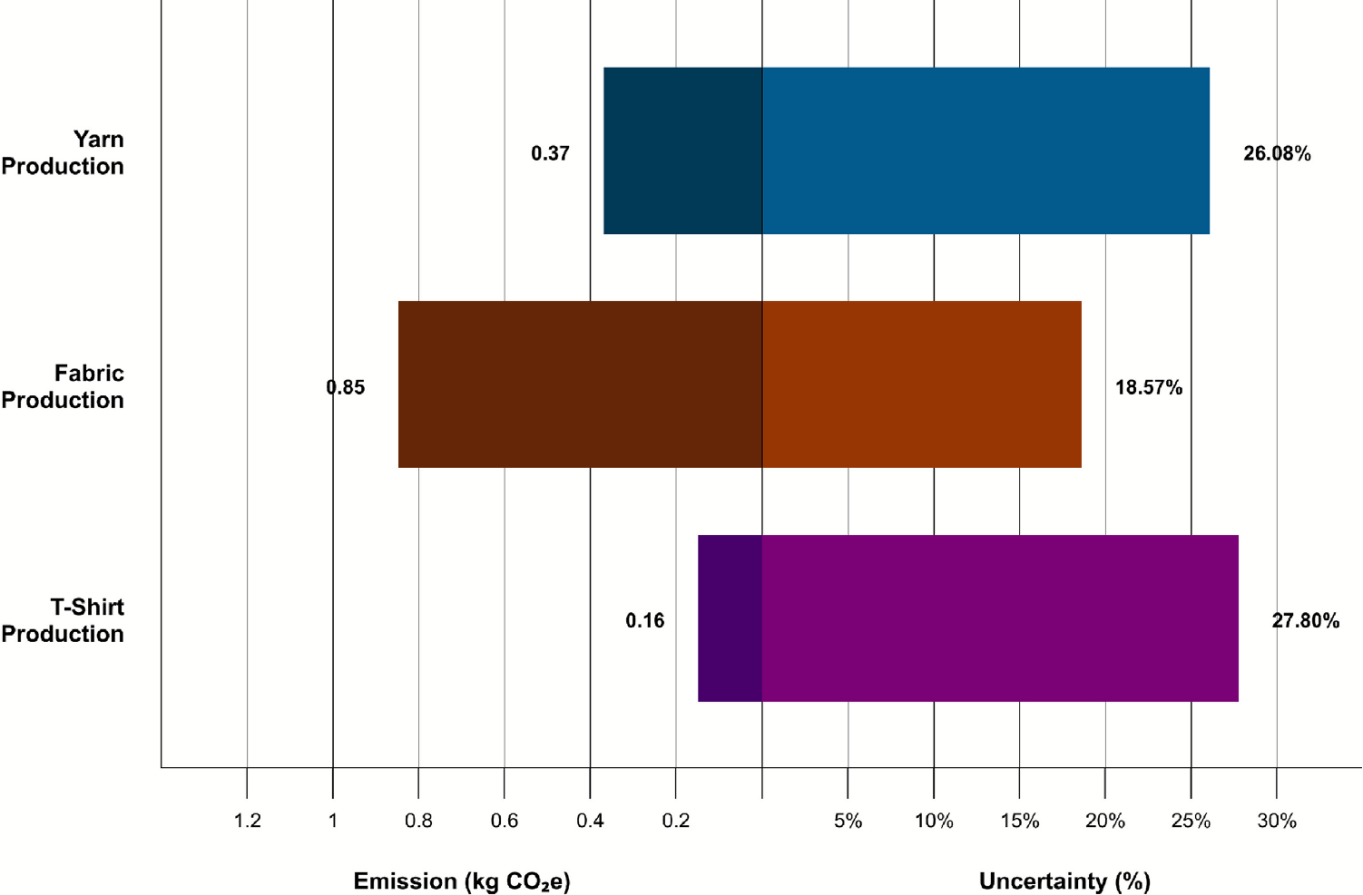
Stage-level contributions to overall emissions and their respective shares of total uncertainty.

Figure 6 illustrates the contrast between absolute emissions and relative uncertainty across the three production stages. The bar lengths show the emission contributions of yarn production, fabric production, and T-shirt assembly, while the overlaid percentages highlight how uncertainty varies in magnitude between stages. Although fabric production contributes the highest share of emissions, yarn and T-shirt production exhibit proportionally higher uncertainty, revealing a mismatch between emission dominance and data reliability.

**Fig. 7:**
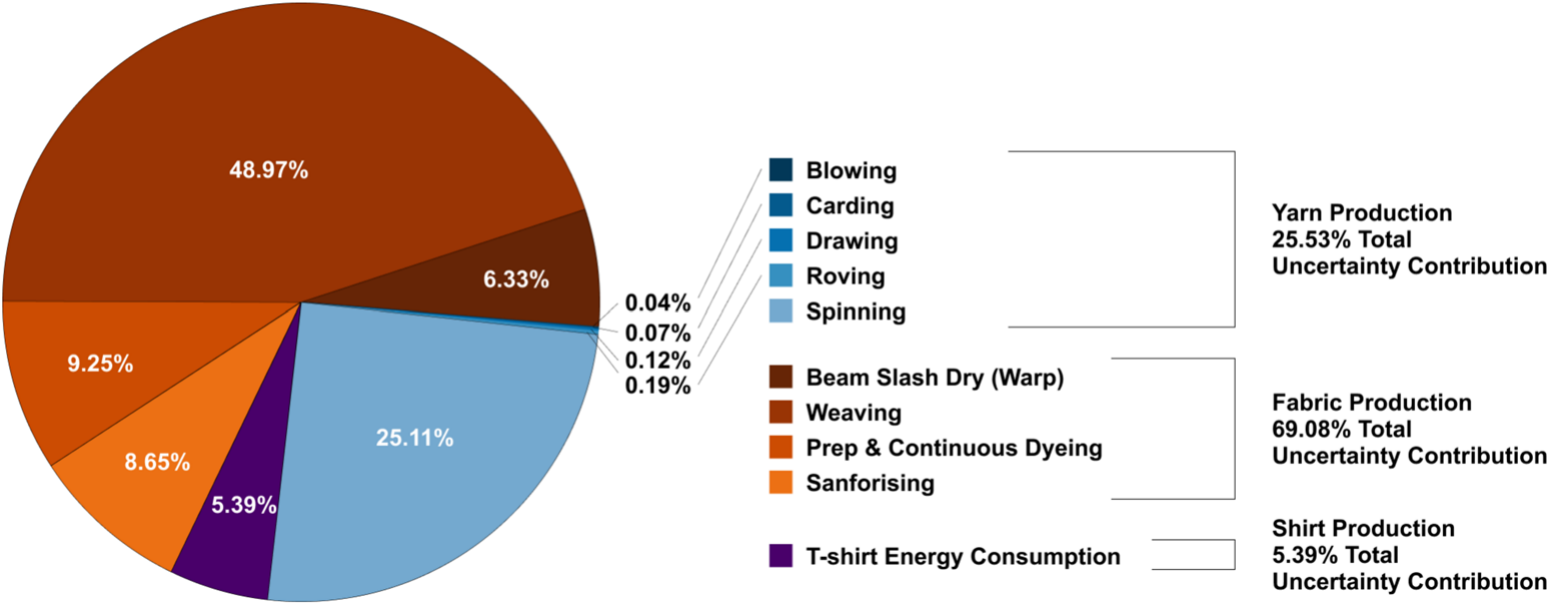
Contributions to total stage-level uncertainty by stage and process.

Using the stage-level uncertainty values, the overall uncertainty for the T-shirt was calculated to be 13.81 %. Fabric production was the dominant source of this uncertainty, contributing 69.08 % of the total. The cradle-to-gate carbon footprint of a single cotton T-shirt was estimated at ${1.3706}\,\hbox {kg}\hbox {CO}_{2}\hbox {e}$ ±0.1892 kg (±13.81 %). Figure 7 presents the percentage contribution of each stage to total uncertainty. Among the individual processes, weaving was the largest contributor at 48.97 %, followed by spinning at 25.11 %. Blowing, carding, drawing, and roving contributed minimally, accounting for 0.04 %, 0.07 %, 0.12 %, and 0.19 %, respectively.

### Technological variability in uncertainty

**Fig. 8:**
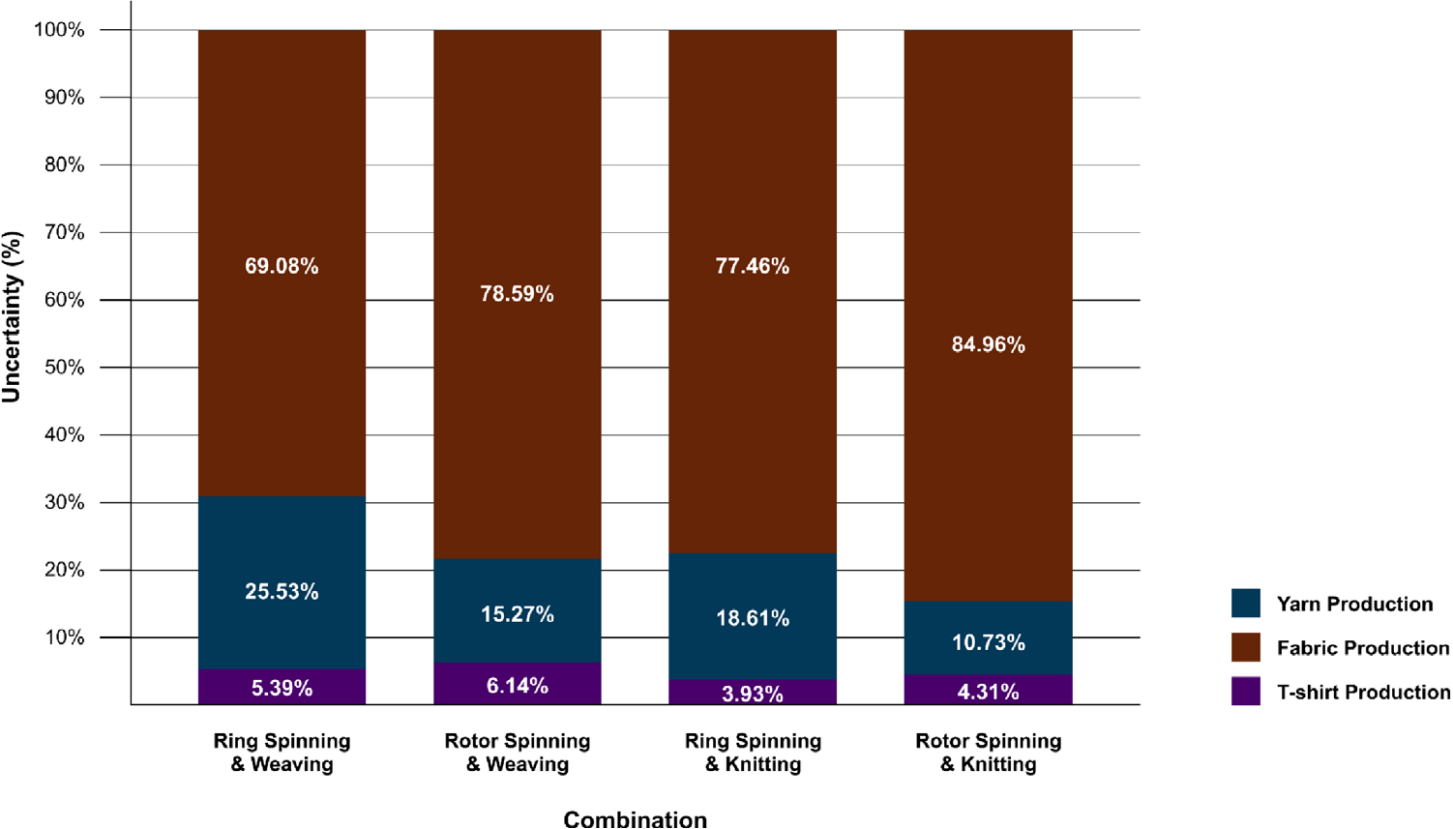
Stage contributions to product-level uncertainty across technology variants.

This analysis is based on a T-shirt manufactured using ring-spun yarn and woven fabric. To assess how technological variation affects uncertainty, three alternative manufacturing configurations were compared: rotor-spun with woven fabric, ring-spun with knitted fabric, and rotor-spun with knitted fabric, and their results are presented in Figure 8. The original ring-spun and woven combination exhibited the lowest overall uncertainty at 13.81 %, followed by rotor-spun and woven at 14.11 % (an increase of 0.30 %), ring-spun and knitted at 14.26 %, and rotor-spun and knitted at 14.69 %. These differences represent only marginal increases in total uncertainty.

Figure 8 also shows that although the overall uncertainty changes slightly, the distribution across stages varies more substantially. Fabric production in the rotor-spun and knitted scenario contributed 84.96 % of the total uncertainty, compared with 69.08 % in the original configuration. The rotor-spun and woven combination showed a contribution of 78.59 %, and the ring-spun and knitted configuration 77.46 %. These findings indicate that rotor spinning and knitting increase the share of uncertainty attributable to the fabric production stage.

### Comparisons with literature

**Fig. 9:**
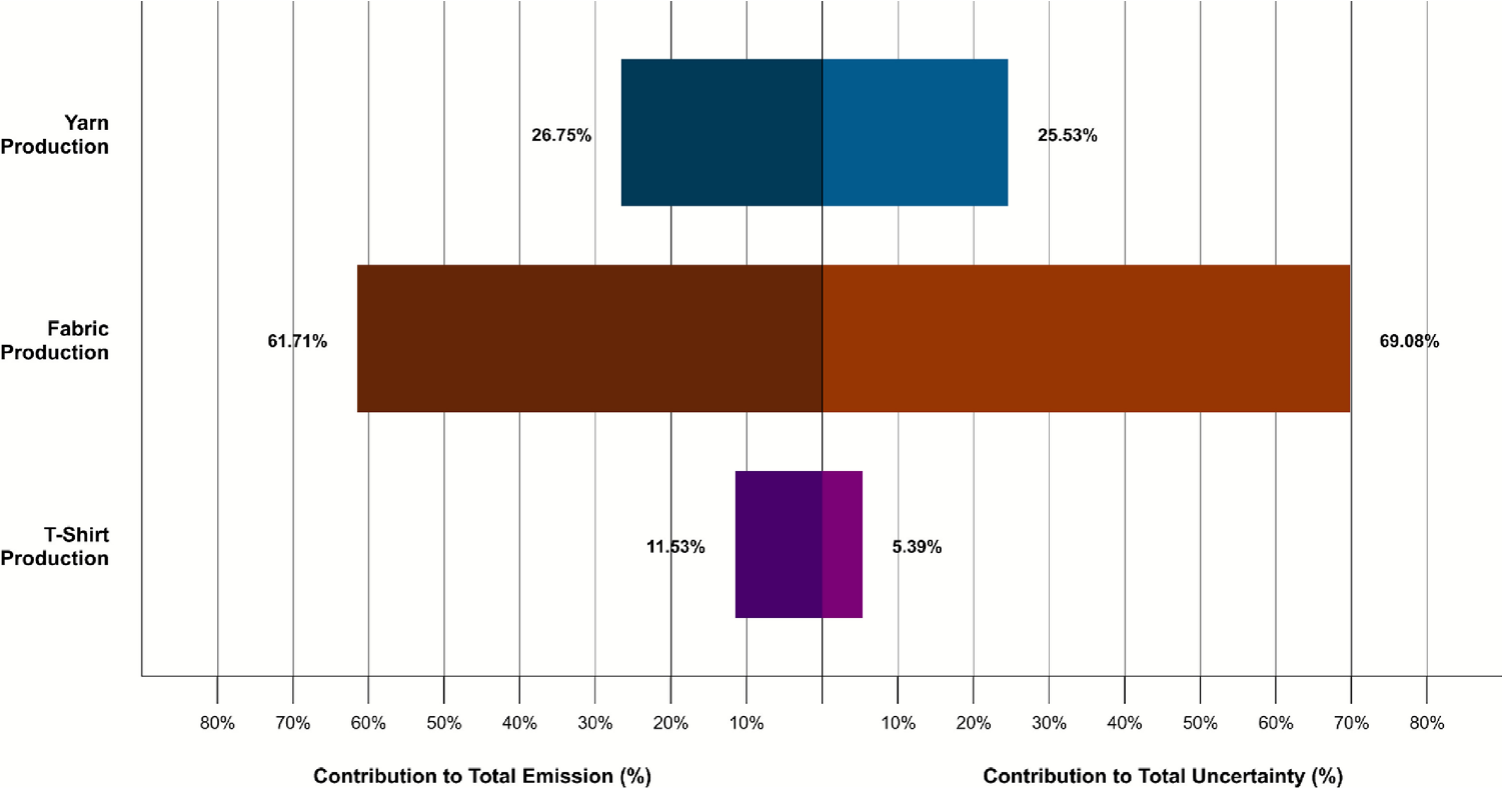
Comparison of percentage contributions to total emissions (left) and total uncertainty (right) by stage for this study.

Figure 9 presents the percentage contribution of each stage to both total emissions and total uncertainty in T-shirt manufacturing. In this analysis, the fabric production stage accounted for approximately 70 % of the overall uncertainty, while yarn production and T-shirt sewing contributed around 25 % and 5 %, respectively. Fabric production also contributed 61.71 % of total emissions, compared with 26.75 % from yarn production and 11.53 % from T-shirt assembly. These results are consistent with previous studies that identify textile manufacturing processes as the dominant source of emissions relative to upstream fibre production^23,31^.

Table 4 summarises findings from previous LCAs of cotton textiles. Barnes et al. found that the consumer use phase contributed the largest share of life cycle impacts, accounting for approximately 82 % of Global Warming Potential (GWP) and 63 % of Acidification Potential (AP). Textile production, which includes yarn and fabric manufacturing, followed with around 18 % of GWP and 27 % of AP, while fibre cultivation contributed roughly 1 % to GWP and 10 % to AP^31^. Jewell et al. similarly identified weaving and dyeing as the highest-impact processes, contributing approximately 50 % of GWP for a knit collared casual shirt, primarily due to electricity consumption and thermal energy demand^23^.

**Table 4:** Comparison of cotton textile LCA findings in literature.

Study	Region/scope	Functional unit	Major contributors
This work	Yarn to sewing	1 T-shirt (150 g)	Fabric production (69 % of uncertainty), yarn (25 %), sewing (5 %); weaving and spinning dominate.
^31^	US, China, India, Turkey, LatAm (cradle-to-grave)	Knit shirt/woven pant	Use phase largest; textile manufacturing second; fibre cultivation lowest.
^23^	Global (China, India, US, Australia)	Knit and woven garments	Textile manufacturing (weaving/dyeing) highest energy/GWP; agriculture lower share.
^19^	Turkey	1 kg fabric	Dyeing/finishing highest; weaving significant.
^32^	Turkey (yarn spinning mills)	1 kg yarn	Spinning energy-intensive vs carding/drawing; open-end reduces energy.

This comparison confirms that textile production is consistently the most emission-intensive and uncertainty-prone stage across regions. However, because this study adopts a cradle-to-gate boundary, its findings diverge from global assessments that include the use and end-of-life phases, where laundering and drying typically emerge as the dominant hotspots.

### Implication for carbon accounting

The findings of this study have several implications for how carbon accounting results are interpreted and applied in decision-making. First, the concentration of uncertainty in a small number of processes, particularly fabric weaving and yarn spinning, indicates that data reliability is uneven across the value chain within a physical-based framework. As a result, cradle to gate carbon footprints should not be presented as single point values but as ranges, consistent with the recommendations of the GHG Protocol^15,30^. Doing so improves transparency and reduces the risk of overconfidence in estimates used for product comparison, policy design, or consumer-facing labelling.

Second, the results highlight both the strengths and limitations of physical-based carbon accounting. This approach provides process-level traceability and enables the identification of specific hotspots for uncertainty reduction. However, its reliability is constrained by the frequent reliance on secondary datasets and generic process inventories. While differences in electricity generation mixes are explicitly accounted for through region-specific emission factors, other sources of heterogeneity become more pronounced when technologies, operational practices, or process configurations differ across regions or facilities but are represented using averaged data. This is particularly relevant for energy-intensive and technology-sensitive stages such as fabric production. Without primary, facility-level data capturing actual process performance, equipment efficiency, and technology choice, physical-based assessments may underrepresent site-specific variability and mischaracterise emissions. Expanding the use of primary data and direct on-site measurements would enhance the credibility, robustness, and decision relevance of carbon accounting for process optimisation and supply chain engagement.

Third, wide uncertainty intervals pose challenges for comparison across studies. Two LCAs may report similar mean values yet differ substantially in confidence ranges, particularly when based on different databases or electricity mixes. This complicates benchmarking and reduces the interpretability of results for both internal decision-making and external disclosure. Greater harmonisation of emission factors and alignment in pedigree matrix scoring practices will be essential if physical-based carbon accounting is to enable meaningful comparisons across textile supply chains.

Finally, for policymakers, recognising that data reliability varies across processes can inform the design of reporting requirements. Targeting high uncertainty stages for stricter verification or disclosure standards, particularly in industrial reporting regimes, may improve the credibility and usefulness of emissions data.

### How to reduce uncertainty

The results from this study, together with findings from other LCAs of the cotton textile chain, indicate that uncertainty is driven less by methodological choice and more by the quality, completeness, and representativeness of the underlying data. It is known that data quality gaps constitute a significant barrier to reliable assessment^31^. Reducing uncertainty can be advanced through the following strategies:

1. **Improving data quality:** Collecting primary data directly from mills and farms, especially for high-impact processes such as weaving, spinning, and dyeing, can reduce dependence on generic datasets. Wider adoption of digital monitoring technologies, including energy meters and water-use sensors, can generate real-time, process-level data that improves the accuracy of LCAs.
2. **Updating and harmonising emission factors:** Outdated emission factors remain major contributors to uncertainty. Regularly updating databases with region-specific values and harmonising emission factors across sources would reduce variability and prevent methodological inconsistencies across studies.
3. **Targeting process-specific hotspots:** As shown in Figure 7, uncertainty is not evenly distributed across the value chain. Prioritising emission- and uncertainty-intensive processes enables researchers, manufacturers, and policymakers to allocate effort and resources where they have the greatest impact.
4. **Using more robust quantitative methods:** Probabilistic techniques, such as Monte Carlo simulation, can better capture the range and distribution of possible outcomes. This is particularly relevant for textile processes where emission intensities vary across technologies, facilities, and regional infrastructures.

These strategies strengthen the robustness of physical-based carbon accounting and also support emerging requirements for industrial disclosure, digital product passports, and supply chain transparency.

## Limitations

This analysis is subject to limitations that influence the accuracy of the results and the interpretation of uncertainty:

- **Data availability:** The study relies on secondary data rather than primary measurements collected directly from facilities. Although secondary data are widely used in product carbon accounting, they introduce representativeness issues, especially for processes such as spinning and weaving, where operational conditions vary significantly across regions and technologies. This affects the precision and completeness indicators in the pedigree matrix.
- **Exclusions from system boundary:** The cradle-to-gate boundary excluded transportation, machinery manufacturing, and auxiliary inputs such as lubricants and packaging. Although these activities usually contribute less to total impacts than the main production processes, their omission may slightly underestimate the overall footprint.
- **Pedigree matrix approach:** The uncertainty analysis relies on pedigree-matrix–based parameter uncertainty, which offers a transparent and practical way to characterise data quality but has recognised limitations. Pedigree scoring involves qualitative judgement, assumes independence between parameters, and does not capture non-linear interactions or fully represent parameter importance. Consequently, uncertainty contributions may not fully reflect the influence of all model parameters, particularly in more complex systems^33^.

To improve the robustness and representativeness of physical-based carbon accounting in future work, these limitations can be mitigated through the following measures:

1. Collecting primary factory-level data for key processes, potentially in collaboration with textile mills.
2. Using hybrid approaches that combine secondary datasets with targeted primary data to reduce reliance on proxies for high-impact stages.
3. Extending the system boundary to include cotton cultivation, use phase, and end-of-life management.

## Conclusion

Physical-based carbon accounting provides valuable process-level insight into the emissions associated with cotton T-shirt manufacturing, but its accuracy remains limited by data quality and representativeness. The use of the pedigree matrix showed that uncertainty is concentrated in a small number of energy-intensive processes, particularly weaving and spinning, where dependence on secondary datasets and generic or outdated emission factors reduces precision. The estimated cradle to gate footprint of ${1.37}\,\hbox {kg}\hbox {CO}_{2}\hbox {e}$ (±13.81 %) reflects both the scale of emissions and the variability embedded in current assessment practices.

These findings underscore the need for improved primary data collection, better alignment between emission factors and actual technologies, and greater harmonisation across databases to reduce uncertainty and improve comparability. Expanding future analyses to include the use and end-of-life phases, testing the influence of yield and electricity assumptions through sensitivity analysis, and integrating hybrid data approaches would also strengthen the robustness of assessment outcomes. By addressing these gaps, physical-based carbon accounting can become more transparent, reliable, and decision-relevant for industry, policymakers, and supply chain stakeholders. Strengthening data quality and uncertainty reporting will also be essential for emerging requirements in industrial disclosure, digital product passports, and sustainability regulation.

## Supplementary Information

Supplementary Information.

## Data Availability

All data supporting the findings of this study are included within the manuscript.
