# Supplementary material for: Uncertainty analysis of physical-based carbon accounting in cotton T-shirt manufacturing
Source: Sci Rep. 2026 Feb 6;16:7586. doi: 10.1038/s41598-026-38773-4 (PMC12936201; doi:10.1038/s41598-026-38773-4)
Supplement: Supplementary file 1 — Supplementary Information. [file 41598_2026_38773_MOESM1_ESM.pdf]

Uncertainty Analysis of Physical-Based Carbon Accounting in Cotton T-shirt Manufacturing

Emmanuel Olugbemi[1] and Natanael Favero Bolson[1*]

[1]School of Engineering, University of Birmingham, Birmingham, B15 2TT, United Kingdom.

*Corresponding author(s). E-mail(s): n.o.faverobolson@bham.ac.uk;
Contributing authors: eoo458@alumni.bham.ac.uk;

Contents

1 Calculation tables **2**

2 Pedigree scores by process **3**

1 Calculation tables

In Table S1, the column *Adjusted Yield* represents the emissions associated with producing 1 kg of finished T-shirt. This accounts for process losses across the value chain. For example, in the case of blowing, the direct emission factor is 0.068 $kgCO_2$/kg of yarn. However, because more than 1 kg of yarn is required to obtain 1 kg of finished garment, the adjusted value increases to 0.078 $kgCO_2$/kg.

The column *Adjusted FU* normalises emissions to the functional unit of the study, which is a 0.15 kg T-shirt. Therefore, the adjusted emission per functional unit is obtained by dividing the adjusted yield by 0.15 kg.

Table S1 Process-level activity data, emission factors, adjusted emissions, and contributions to the total cradle-to-gate footprint for one 150 g cotton T-shirt.

Stage	Process	AD [1] (kW h/kg)	EF [2] ($kgCO_2$/kWh)	Emissions ($kgCO_2$/kg)	Adj. Yield[1] ($kgCO_2$/kg)	Adj. FU[2] ($kgCO_2$/kg)	Share (%)
**Yarn Production**	Blowing	0.0832	0.8225	0.068	0.078	0.0120	0.88
	Carding	0.1068	0.8225	0.088	0.100	0.0155	1.13
	Drawing	0.1417	0.8225	0.116	0.132	0.0205	1.50
	Roving	0.1770	0.8225	0.145	0.165	0.0256	1.87
	Spinning	2.0222	0.8225	1.658	1.890	0.2930	21.37
Subtotal						0.3667	26.75
**Fabric Production**	Beam Slash Dry (Warp)	0.6144	0.8225	0.504	0.560	0.0868	6.33
	Weaving	2.8972	0.8225	2.376	2.640	0.4092	29.85
	Prep & Continuous Dyeing	1.2592	0.8225	1.033	1.148	0.1779	12.98
	Sanforising	1.2186	0.8225	0.999	1.110	0.1720	12.55
Subtotal						0.8459	61.71
**T-shirt Production**	Energy Consumption	1.5180[3]	0.8220[4]	1.020	1.020	0.1581	11.53
Subtotal						0.1581	11.53
**Total**						**1.3706**	**100.00**

[1] adjusted to the material yield
[2] adjusted to the functional unit (FU)

2 Pedigree scores by process

Table S2 Pedigree scoring of activity data (AD) and emission factors (EF) for selected processes.

Process	Type	Unit	P	C	T	G	R	Source
**Yarn Production**								
Blowing	AD	kW h/kg	1.10	1.05	1.20	1.02	1.20	[1]
	EF	$kgCO_2/kWh$	1.00	1.00	1.00	1.02	1.00	[2]
Carding	AD	kW h/kg	1.10	1.05	1.20	1.02	1.20	[1]
	EF	$kgCO_2/kWh$	1.00	1.00	1.00	1.02	1.00	[2]
Drawing	AD	kW h/kg	1.10	1.05	1.20	1.02	1.20	[1]
	EF	$kgCO_2/kWh$	1.00	1.00	1.00	1.02	1.00	[2]
Roving	AD	kW h/kg	1.10	1.05	1.20	1.02	1.20	[1]
	EF	$kgCO_2/kWh$	1.00	1.00	1.00	1.02	1.00	[2]
Spinning	AD	kW h/kg	1.10	1.05	1.20	1.02	1.20	[1]
	EF	$kgCO_2/kWh$	1.00	1.00	1.00	1.02	1.00	[2]
**Fabric Production**								
Beam Slash Dry (Warp)	AD	kW h/kg	1.10	1.05	1.20	1.02	1.20	[1]
	EF	$kgCO_2/kWh$	1.00	1.00	1.00	1.02	1.00	[2]
Weaving	AD	kW h/kg	1.10	1.05	1.20	1.02	1.20	[1]
	EF	$kgCO_2/kWh$	1.00	1.00	1.00	1.02	1.00	[2]
Prep & Continuous Dyeing	AD	kW h/kg	1.10	1.05	1.20	1.02	1.20	[1]
	EF	$kgCO_2/kWh$	1.00	1.00	1.00	1.02	1.00	[2]
Sanforising	AD	kW h/kg	1.10	1.05	1.20	1.02	1.20	[1]
	EF	$kgCO_2/kWh$	1.00	1.00	1.00	1.02	1.00	[2]
**T-shirt Production**								
Energy consumption	AD	kW h/kg	1.10	1.05	1.20	1.02	1.00	[3]
	EF	$kgCO_2/kWh$	1.10	1.00	1.10	1.02	1.00	[4]

P = precision, C = completeness, T = temporal representativeness, G = geographical representativeness, R = technological representativeness.
India EF rows use national grid CO_2 intensities for the stated period; sewing (T-hirt production) EF uses China-specific values.
Uncertainty factors follow the GHG Protocol pedigree matrix framework applied in this study.

References

[1] Jewell J, Koffler C, Murphy S. LCA Update of Cotton Fiber and Fabric Life Cycle Inventory. Thinkstep; 2017.

[2] SFC. India Default GHG Emission Values. Smart Freight Centre India; 2025.

[3] Zhang Y, Liu X, Xiao R, Yuan Z. Life cycle assessment of cotton T-shirts in China. The International Journal of Life Cycle Assessment. 2015 7;20(7):994–1004. https://doi.org/10.1007/s11367-015-0889-4.

[4] Zhang X, Zhu Q, Zhang X. Carbon Emission Intensity of Final Electricity Consumption: Assessment and Decomposition of Regional Power Grids in China from 2005 to 2020. Sustainability. 2023 6;15(13):9946. https://doi.org/10.3390/su15139946.
